# XIST promotes cell proliferation and invasion by regulating miR-140-5p and SOX4 in retinoblastoma

**DOI:** 10.1186/s12957-020-01825-8

**Published:** 2020-03-03

**Authors:** Yuhui Wang, Dahong Sun, Ying Sheng, Hong Guo, Fanchun Meng, Tingting Song

**Affiliations:** 1 Department of Clinical Laboratory, Affiliated Hospital of Beihua University, Jilin, 132011 China; 2 Department of Pediatrics, The Third People’s Hospital of Qingdao, Qingdao, 266041 China; 3 Health Management Center, The People’s Hospital of Zhangqiu Area, Jinan, 250200 China; 4 Delivery Room, The People’s Hospital of Zhangqiu Area, Jinan, 250200 China; 5 No. 2 Department of Oncology, Qingdao Central Hospital, Qingdao Tumor Hospital, 127 Siliu South Road, Shibei District, Qingdao, 266042 China

**Keywords:** Retinoblastoma, XIST, miR-140-5p, SOX4

## Abstract

**Background:**

Retinoblastoma (RB) is the most common intraocular malignancy in children. Long non-coding RNA X-inactive specific transcript (lncRNA XIST) has been reported to be associated with RB, but research on the mechanism of XIST is not well studied.

**Methods:**

Expressions of XIST, microRNA-140-5p (miR-140-5p), and sex-determining region Y-related high-mobility group box 4 (SOX4) were analyzed by qRT-PCR or Western blot. Relationships of XIST, SOX4, and miR-140-5p were evaluated by dual-luciferase reporter assay and Spearman’s analysis. Cell Counting Kit-8 (CCK-8) and Transwell assay were performed to assess the function of XIST on RB cell proliferation and invasion.

**Results:**

In RB tissues, XIST and SOX4 expressions were obviously increased, but the miR-140-5p expression was markedly reduced. XIST expression was positively related to SOX4 expression while negatively correlated with miR-140-5p expression, and negative correlation was exhibited between miR-140-5p and SOX4 expression in RB tissues. XIST was confirmed to directly bind to miR-140-5p, and SOX4 was one target of miR-140-5p. XIST knockdown could impede RB cell proliferation and invasion, while miR-140-5p inhibition reversed the effects. In addition, XIST overexpression or miR-140-5p inhibition could abrogate the inhibition of SOX4 silencing on cell proliferation and invasion of RB cells.

**Conclusions:**

XIST was obviously increased in RB tissues and cells, and XIST inhibition repressed the proliferation and invasion of RB cells by miR-140-5p/SOX4 axis, which may provide new understandings of the XIST molecular mechanism in RB.

## Background

Retinoblastoma (RB) is the most common intraocular malignancy in children and rare in adults, with poor prognosis, which seriously affects the vision of children and even endangers life [1]. The etiology of RB is extremely complicated and remains unknown, which may be related to heredity and virus infection [2]. RB accounts for about 3% of childhood malignancies; in order to improve the survival rate and reduce the rate of eyeball removal, the treatment methods are constantly updated and developed [3]. At present, the main treatment methods are chemotherapy, radiotherapy, surgery, physical therapy, photochemotherapy, gene therapy, and so on [4]. However, the prognosis in life and vision is still not satisfactory. Therefore, it is imperative to explore the pathological mechanism of RB to identify novel biomarkers for RB diagnosis and treatment.

Increasing researches have confirmed that lncRNAs are associated with RB tumorigenesis and progression. For instance, plasmacytoma variant translocation 1 (PVT1) promoted RB progression by its upregulation [5]. Maternally expressed gene 3 (MEG3) was downregulated and suppressed the RB progression [6]. The study of Zhang et al. suggested that H19 was a decreased expression and suppressed RB progression [7], while Li et al. found that H19 was upregulated in RB and may act as an oncogenic function in RB progression [8]. X-inactive specific transcript (XIST), a widely studied lncRNA, has been reported that it was dysregulated and functioned as an oncogene in RB [9, 10]. However, our knowledge about detailed mechanisms for XIST in RB is still insufficient.

MiRNAs with about 22 nucleotides length were dysregulated as tumor suppressor or oncogene under certain circumstances in human cancers [11]. Accumulating studies reported that lncRNAs were involved in cancer progression through miRNA-mediated gene regulation by sponging miRNA [12]. miR-140-5p, a miRNA, has been reported as a suppressor in several tumors, like colorectal carcinoma [13], gastric cancer [14], and oral squamous cell carcinoma [15]. Several studies confirmed miR-140-5p also inhibited RB progression as a tumor suppressor [16–18]. However, the specific regulatory mechanisms of it, especially its related lncRNA-miRNA-mRNA regulatory networks, are still limited and need to be further explored.

SOX4, one of the sox transcription factors, has been proven as an oncogene and correlated with tumor progression and development [19, 20]. It has been reported that lots of miRNAs could modulate SOX4 in human cancers [19]. Kooi et al. demonstrated SOX4 as novel RB driver candidates by somatic copy number alteration profiling [21]. At present, research on the function of SOX4 in RB has not been studied still in a deepgoing way by now.

Thereby, XIST, miR-140-5p, and SOX4 expressions and possible mechanisms were explored in RB in our study. We found that XIST and SOX4 expressions were increased while miR-140-5p was decreased both in RB tissues and cells. In addition, XIST knockdown suppressed proliferation and invasion of RB cells by miR-140-5p/SOX4 axis, indicating XIST might be a potential biomarker for RB treatment and diagnosis.

## Materials and methods

### Tissue specimens

Eight normal retinas and 20 RB tissues were obtained from the Affiliated Hospital of Beihua University. No chemotherapy and local radiotherapy were received before the operation for all patients. This study obtained the Ethical Review of Affiliated Hospital of Beihua University and informed consent of all patients or guardians.

### Cell culture

ARPE-19 (human retinal epithelial cells) and Y79, Weri-Rb1, SO-Rb50, and HXO-RB44 (human RB cell lines) which were obtained from ATCC were cultured in DMEM (Life Technologies, Carlsbad, USA) media containing 10% fetal bovine serum (FBS, Gibco, USA). Cells were cultured with 5% CO_2_ at a 37 °C incubator.

### Cell transfection

Si-XIST (small interference RNA of XIST), pc-XIST (XIST-overexpressing plasmid), pc-SOX4 (SOX4-overexpressing plasmid), and miR-140-5p mimic and inhibitor were constructed from GenePharma (Shanghai, China). Lipofectamine 2000 (Invitrogen, USA) was for transient transfection of constructs into cells with the manufacturer’s indication.

### CCK-8 assay

Cell proliferation reagent CCK-8 (Roche, Basel, Switzerland) was used to detect cell proliferation in CCK-8 assay. Each group cell was seeded in a 96-well plate (Corning, NY, USA). At 1, 2, 3, and 4 d after the transfection of each group, 10 μL CCK-8 reagents were added to each well. Subsequently, the 96-well plate was incubated at 37 °C for 2 h. At last, the values of spectrophotometric absorbance at 450 nm were detected and recorded.

### Cell invasion assays

Cell invasion was detected using Transwell chamber. Briefly, transfected Y79 were seeded in the upper chambers with a Matrigel-coated and serum-free medium, meanwhile 700 μL medium containing 15% FBS was inserted into the lower chamber. After 48 h incubation, the outer cells were fixed and then stained with 20% Giemsa solution. Five random views were selected for cell counting with an inverted microscope (CK2, Olympus).

### Dual-luciferase activity assay

Luciferase reporter vectors including wild-type and mutant-type (XIST-wt, XIST-mut, SOX4-wt, and SOX4-mut) were amplified and constructed into the pGL3-basic vector (Promega, USA) respectively. RB cells were transfected with miR-140-5p mimics or NC along with the above constructed vectors. After transfection for 48 h, Dual-Luciferase Reporter Assay kit (Promega, USA) was used to measure firefly luciferase activity.

### Quantitative real-time PCR

Total RNA was extracted by TRIzol reagent (Invitrogen, USA), and reverse transcription action was performed by Reverse Transcription Kit (Takara, Japan). qRT-PCR reactions were performed with SYBR Green (Takara) on Bio-Rad CFX96TM System (CA, USA). Primers were listed in Table 1. The expression levels were calculated by the 2^−ΔΔCt^ method and normalized by U6 and glyceraldehyde phosphate dehydrogenase (GAPDH) expression.

**Table 1:** Primer sequences for real-time fluorescence quantification PCR

Gene name	Primer sequences (5′-3′)
GAPDH	F: ACGCTGCATGTGTCCTTAG R: GAGCCTCTTATAGCTGTTTG
U6	F: CTCGCTTCGGCAGCACA R: AACGCTTCACGAATTTGCGT
XIST	F: AATGACTGACCACTGCTGGG R: GTGTAGGTGGTTCCCCAAGG
miR-140-5p	F: CAGTGGTTTTACCCTATGGTAG R: ACCATAGGGTAAAACCACTGTT
SOX4	F: GGCCTGTTTCGCTGTCGGGT R: GCCTGCATGCAACAGACTGGC

### Western blot

Total proteins were extracted by RIPA reagent (Beyotime, China) and separated using 10% SDS-PAGE. Subsequently, the bands were transferred onto polyvinylidene difluoride (PVDF) membrane (Sigma-Aldrich, USA) and probed with primary antibodies (SOX4 and GAPDH antibodies, 1:1000 dilution) at 4 °C overnight. Then, membranes were incubated for another 2 h with secondary antibodies conjugated to HRP at room temperature. All antibodies were purchased from Abcam (Cambridge, UK). The bands were visualized by enhanced chemiluminescence (ECL) method.

### Statistical analysis

Data are presented as the mean ± standard deviation (SD). The differences between the groups were compared using Graph Prism 5.0 software (San Diego, CA, USA) with a one-way analysis of variance (ANOVA) or Student *t* test. *P <* 0.05 indicated a statistically significant result in comparison.

## Results

### XIST expression was increased in RB tissues and cells

**Fig. 1:**
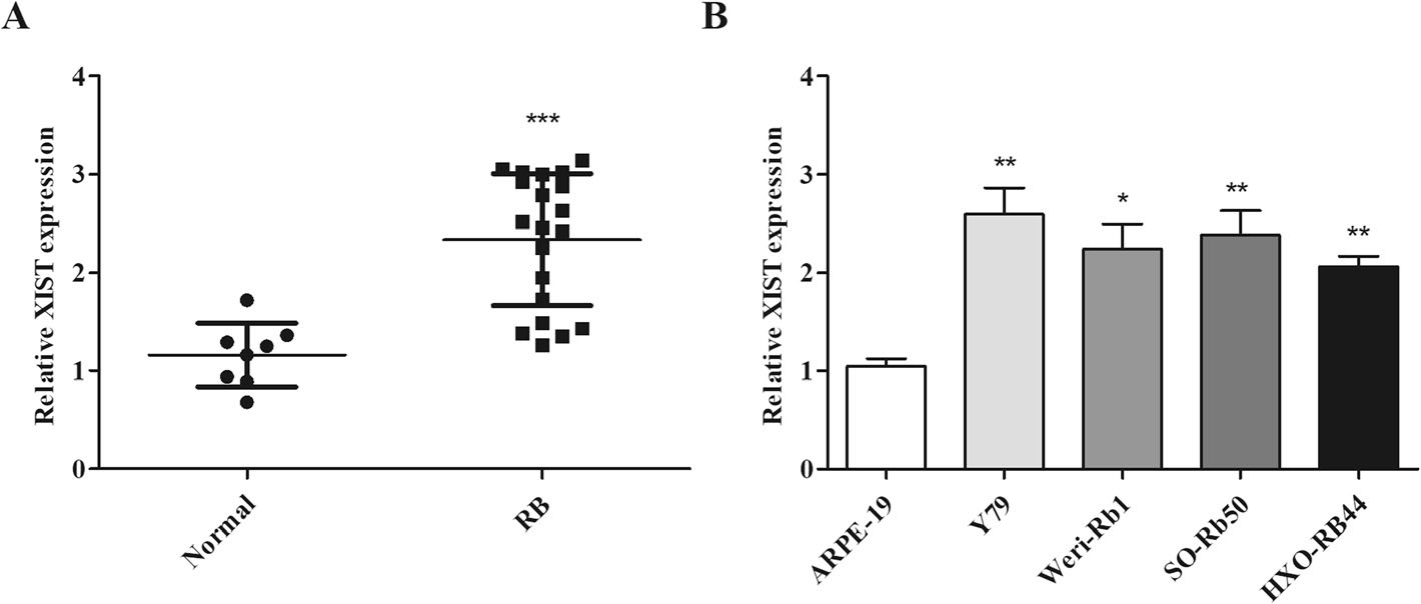
Expression of XIST was examined in RB tissues and cells. **a** Expression of XIST was significantly upregulated in RB tissues. **b** XIST expression was remarkably upregulated in RB cells compared to human retinal epithelial cells. *P < 0.05, **P < 0.01, and ***P < 0.001, compared with normal tissues or the ARPE-19 group

Firstly, we analyzed the XIST expression in RB tissues and found XIST expression was obviously increased in RB tissues versus normal tissues (Fig. 1a). Similarly, an increasing expression of XIST was observed in RB cells (Y79, Weri-Rb1, SO-Rb50, and HXO-RB44) compared to ARPE-19 (human retinal epithelial cells) (Fig. 1b). Among them, the highest XIST expression was found in Y79 cells, so we chose Y79 cells for follow-up experiments (Fig. 1b). These data suggested that dysregulation of XIST may be associated with RB progression.

### XIST enhances Y79 cell proliferation and invasion

**Fig. 2:**
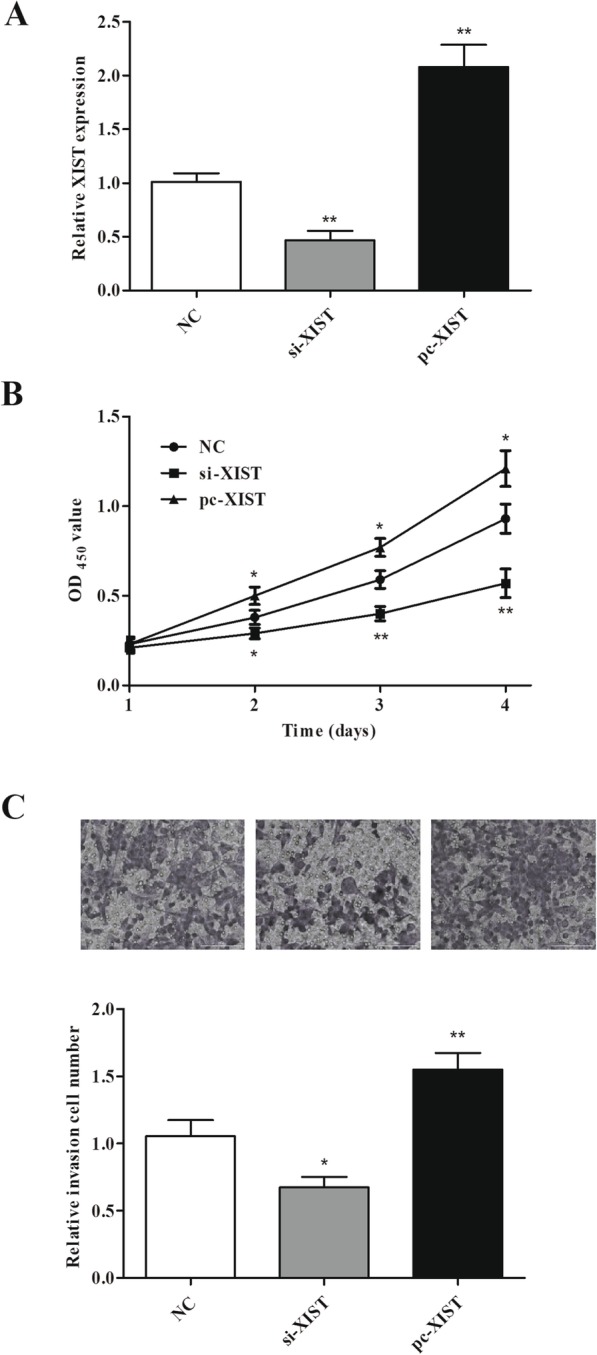
XIST promoted cell proliferation and invasion in Y79 cells. **a** XIST expression was decreased or increased in cells after si-XIST or pc-XIST was transfected. **b** XIST knockdown or overexpression regulated cell proliferation of Y79 cells. **c** Cell invasion of Y79 cells was regulated by XIST knockdown or overexpression. *P < 0.05 and **P < 0.01, compared with the NC group

To detect roles of XIST in RB, si-XIST, pc-XIST, or NC scramble was transfected into Y79. Compared with NC, XIST expression was obviously reduced and enhanced in Y79 cells transfected with si-XIST and pc-XIST, respectively (Fig. 2a). Cell proliferation was confirmed to be restrained in Y79 cells transfected with si-XIST and promoted by pc-XIST in CCK-8 assay (Fig. 2b). Moreover, the Transwell assay showed that cell invasion was also inhibited by XIST knockdown and promoted by XIST overexpression (Fig. 2c). Thus, we suspected that XIST may play a potential carcinogenesis in RB.

### miR-140-5p directly binds to and negatively correlated with XIST

**Fig. 3:**
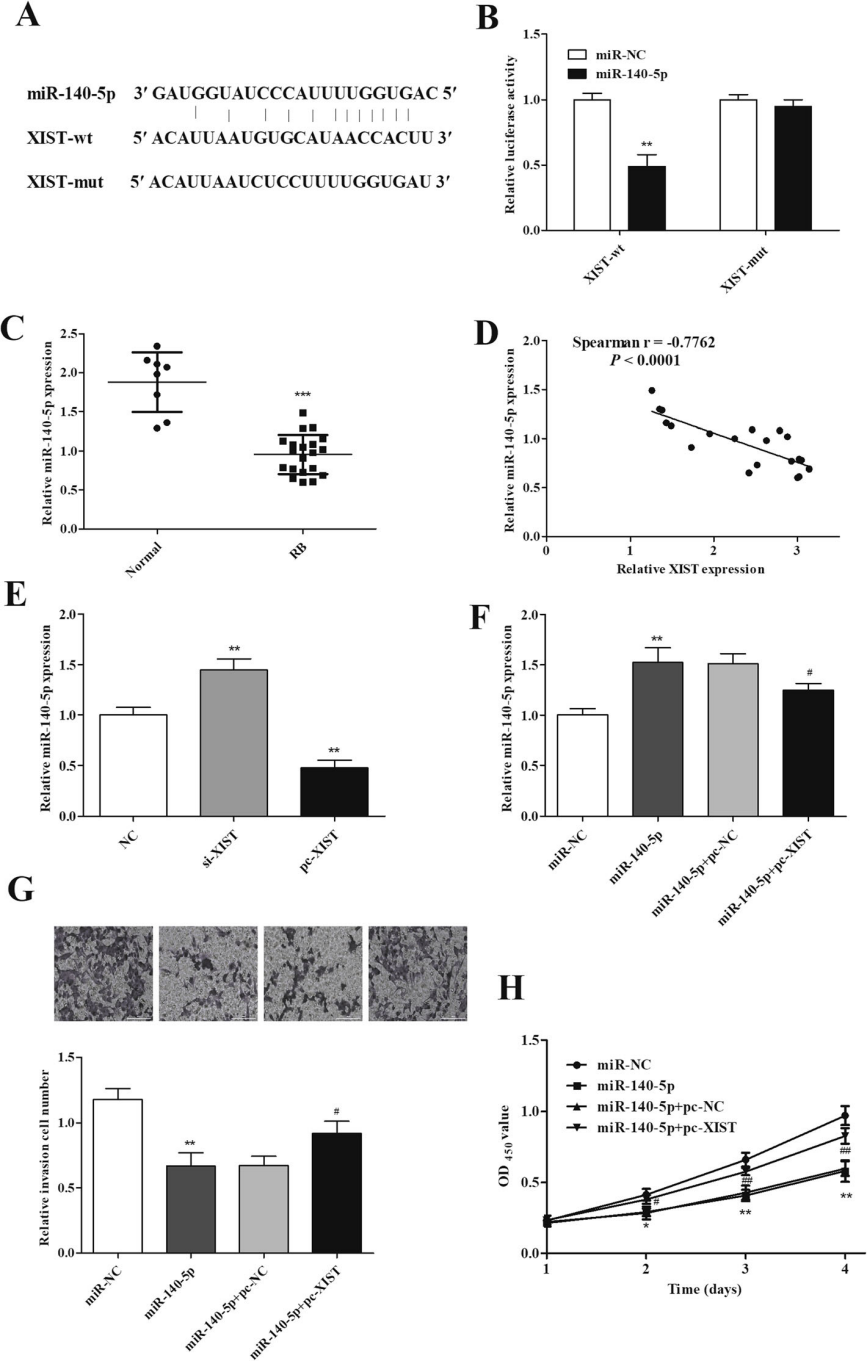
XIST directly binds to miR-140-5p. **a** Predicted binding site between miR-140-5p and XIST. **b** The luciferase reporter assay demonstrated that XIST directly targeted miR-140-5p in Y79 cells. **c** Expression of miR-140-5p was decreased in RB tissue. **d** Correlation analysis between XIST and miR-140-5p in RB tissues. **e** XIST notably inhibits miR-140-5p expression in Y79 cells. **f** Expression of miR-140-5p in Y79 cells transfected with miR-140-5p mimics or miR-140-5p mimics + pc-XIST. **g, h** Repressive effects of miR-140-5p on cell invasion and proliferation of Y79 cells were attenuated by XIST overexpression. *P < 0.05, **P < 0.01, ^*#*^*P <* 0.05, and ^*##*^*P <* 0.01, compared with the miR-NC, miR-140-5p+pc-NC, normal, or NC group

To investigate the regulatory mechanism of XIST in RB, prediction of target miRNA was performed by bioinformatics analysis. XIST is predicted to have potential binding sites with miR-140-5p (Fig. 3a). We performed the luciferase activity assays in order to verify the prediction. Luciferase activity was obviously reduced in the cells transfected with XIST-wt and miR-140-5p mimics compared with other groups (Fig. 3b). In addition, miR-140-5p expression was lower and inversely related to XIST expression in RB tissues (Fig. 3c, d). miR-140-5p expression was reduced after XIST overexpression while reversed by XIST knockdown in Y79 cells (Fig. 3e). To further confirm their relation, miR-140-5p mimics were transfected into Y79 cells along with pc-XIST. The expression of miR-140-5p was increased by its mimics while reduced in the presence of pc-XIST (Fig. 3f). In a rescue experiment, XIST overexpression also attenuated the inhibition of miR-140-5p mimics on cell proliferation and invasion (Fig. 3g, h). Collectively, the results suggested that XIST may promote RB progression by targeting miR-140-5p.

### SOX4 is a direct target of miR-140-5p

**Fig. 4:**
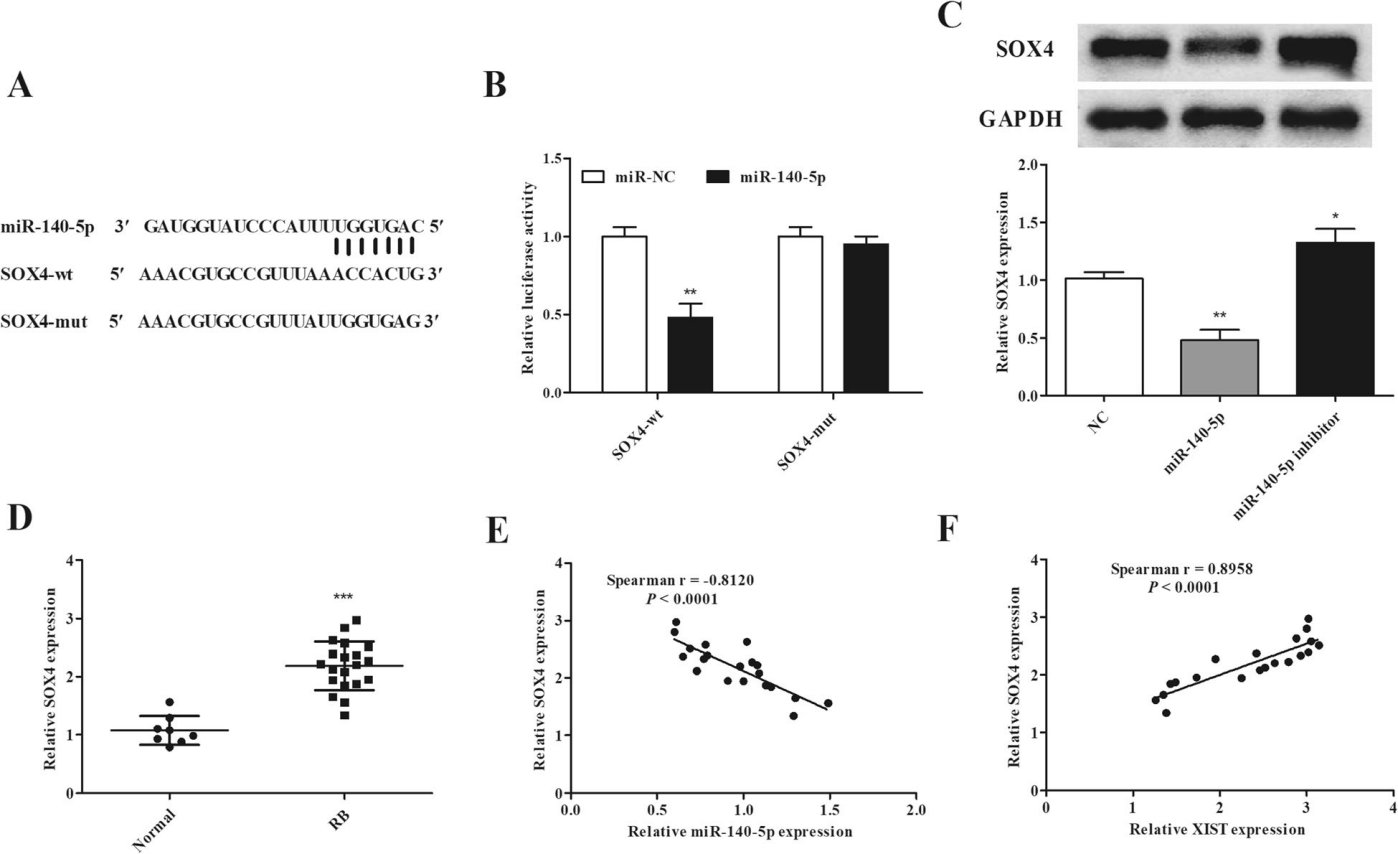
miR-140-5p directly targets SOX4 in Y79 cells. **a** Putative target sites between miR-140-5p and SOX4. **b** Luciferase reporter assay demonstrated that miR-140-5p directly targeted SOX4. **c** miR-140-5p inhibited SOX4 expression levels in Y79 cells. **d** SOX4 mRNA expression was enhanced in RB tissues. **e** The correlation between miR-140-5p and SOX4 in RB tissues. **f** The correlation between XIST and SOX4 in RB tissues. *P < 0.05, **P < 0.01, and ***P < 0.001, compared with the miR-NC, NC, or normal group

We used the TargetScan software to predict miR-140-5p target genes, and SOX4 has binding sites of miR-140-5p (Fig. 4a). Luciferase activity was obviously reduced in Y79 cells with miR-140-5p overexpression and SOX4-wt transfection rather than others (Fig. 4b). Furthermore, SOX4 expressions both in mRNA and protein were downregulated when miR-140-5p was upregulated, while it increased by the inhibition of miR-140-5p in Y79 cells (Fig. 4c). In addition, SOX4 was upregulated in RB tissues versus normal tissues (Fig. 4d), and in RB tissues, it had a negative relationship with miR-140-5p expression (Fig. 4e). Inversely, XIST and SOX4 expression exhibited a positive correlation in RB tissues (Fig. 4f). On the basis of the above results, we speculated that SOX4 may contribute to RB cell proliferation and invasion via XIST/miR-140-5p.

### XIST/miR-140-5p axis promotes cell proliferation and invasion through SOX4

**Fig. 5:**
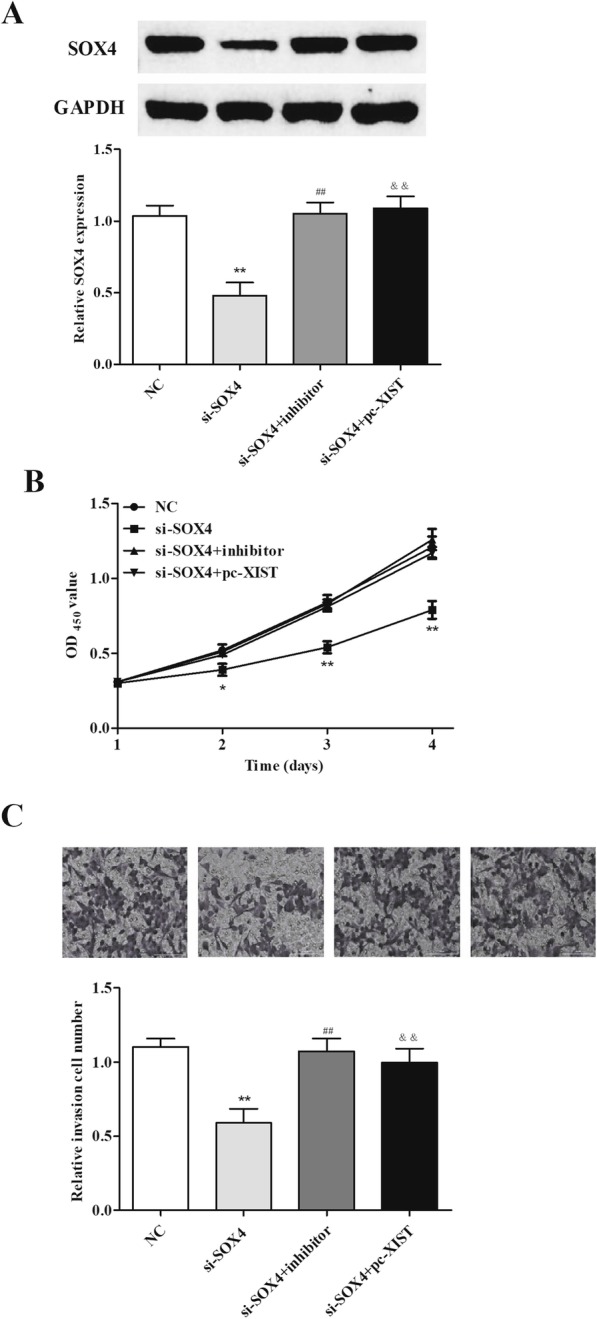
XIST/miR-140-5p may regulate cell proliferation and invasion through SOX4 in Y79 cells. **a** SOX4 expression was repressed by si-SOX4, and it was attenuated by miR-140-5p inhibitor or XIST overexpression. **b**, **c** SOX4 downregulation had a suppressed effect on cell proliferation and invasion of Y79 cells, and it was offset by miR-140-5p inhibitor or XIST overexpression. *P < 0.05, **P < 0.01, ^##^*P <* 0.01, and ^&&^*P <* 0.01, compared with the NC or si-SOX4 group

To test whether XIST can regulate SOX4 via miR-140-5p in RB, we performed qRT-PCR and Western blot assay. We knocked down SOX4 expression in RB cells using si-RNA. Transfection with si-SOX4 significantly could decrease SOX4 expression, and si-SOX4 and miR-140-5p inhibitor or pc-XIST co-transfection increased SOX4 expression (Fig. 5a). Furthermore, the obvious rescue of miR-140-5p inhibitor or pc-XIST on the proliferation and invasion ability induced by si-SOX4 in RB cells were observed in Transwell and CCK-8 assays (Fig. 5b, c). These results indicate that XIST may promote cell proliferation and invasion through the miR-140-5p/SOX4 axis to facilitate RB progression.

## Discussion

RB is the most frequent intraocular cancer of childhood with highly aggressive and metastatic but a rare disease (about 4% of all pediatric malignancies) [22]. Interestingly, increasing studies demonstrated that lncRNAs play important roles in promoting or suppressing RB progression, metastasis, diagnosis, and prognosis, such as HOTAIR, NEAT1, XIST, MEG3, and H19 [23]. XIST, a widely studied lncRNA, is dysregulated and serves as a potential biomarker in multiple cancers [24], but research on the mechanism of XIST is not well studied in RB. In our study, we explored the roles of XIST and its potential regulatory mechanisms on RB progression. Results revealed that XIST was highly expressed in RB; we proposed it might be served as an oncogene in RB. Functionally, XIST inhibition impeded RB cell proliferation and invasion. Meanwhile, XIST was found to directly bind and negatively regulate miR-140-5p to affect SOX4, thereby promoting RB cell proliferation and invasion capability.

XIST has been reported to be aberrantly expressed and closely related to the progression of various cancers. In pancreatic cancer, XIST contributes to improve cell migration, invasion, and EMT capacities by acting as a miR-429 molecular sponge to regulate ZEB1 expression [25]. Another study has verified that the progression of esophageal cancer was promoted by XIST-regulated miR-494/CDK6 axis to activate the JAK2/STAT3 signal pathway [26]. In thyroid cancer, the XIST/miR-34a axis could promote cell proliferation and tumor growth by regulating MET-PI3K-AKT signaling [27]. Tumor metastasis of colorectal cancer was promoted by the acceleration of the XIST/miR-137/EZH2 axis on cell migration and invasion [28]. Nevertheless, there is no extensive data explaining the function of XIST in RB, except for the finding that XIST promotes the EMT of RB by modulating miR-101 and conduces to the progression of RB by regulating miR-124/STAT3 axis [9, 10]. All aforementioned findings are in accordance with our results that XIST might serve as an oncogene in RB progression and XIST inhibition could impede RB cell proliferation and invasion.

Additionally, we excavated the specific mechanism of XIST in RB. LncRNAs are reported as miRNAs sponges which can target downstream genes and play vital roles in various cancers progression [29]. A previous study confirmed that in cervical cancer, XIST promoted cell proliferation and suppressed cell apoptosis via miR-140-5p [30]. Moreover, miR-140-5p has been confirmed to inhibit cell proliferation, migration, and invasion while inducing cell apoptosis in RB [16, 17]. Thus, we proposed that XIST promoted RB cell proliferation and invasion by regulating miR-140-5p. We performed the luciferase reporter assays and confirmed miR-140-5p could directly target XIST. miR-140-5p expression was increased, and a negative association was observed with XIST in RB tissues. Furthermore, in RB cells, miR-140-5p expression was significantly increased by XIST inhibition while decreased by XIST overexpression. Meanwhile, miR-140-5p expression was increased by its mimics while reduced in the presence of XIST overexpression. Finally, XIST overexpression partially abolished the suppression effect of miR-140-5p overexpression on RB cell proliferation and invasion. In this way, XIST contributed to RB progression by negatively regulating miR-140-5p.

Subsequently, SOX4 was predicted as one downstream target of miR-140-5p by biological analysis. In malignant melanoma [31] and colorectal carcinoma [13], miR-140-5p was confirmed to be suppressed cell proliferation and invasion by inhibiting SOX4 expression. Here, we found that SOX4 was upregulated, and its expression has a positive correlation with XIST expression while it was negatively related to miR-140-5p in RB tissues. SOX4 silencing could inhibit RB cell proliferation and invasion, suggesting it may serve as a tumor promoter in RB. More importantly, the inhibition of SOX4 knockdown in cell proliferation and invasion was abolished by miR-140-5p downregulation or XIST upregulation. Overall, our conclusions illustrated that XIST could promote the process of cell proliferation and invasion by modulating miR-140-5p/SOX4 axis, thus accelerating RB progression.

Collectively, we suggested XIST might serve as an oncogene in RB progression by miR-140-5p/SOX4 axis, but still, some limitations are present. First, the function of XIST was not investigated in vivo. Thus, animal experiments (overexpression or silencing) should be carried out. Moreover, we only had a preliminary research on the correlation of XIST and miR-140-5p because of the experimental conditions. Subsequent RNA pull-down or RNA immunoprecipitation experiment should be conducted in cells. Finally, other possible mechanisms of XIST in RB should be explored, such as the effect on cell migration.

## Conclusions

In a word, XIST is closely related to the occurrence and development of RB. XIST inhibition suppressed cell proliferation and invasion of RB cells by the miR-140-5p/SOX4 axis. Therefore, targeting XIST has great significance in the treatment of RB. However, there are many complicated regulatory pathways involving lncRNAs and miRNAs, and it is necessary to clarify specific signal transduction mechanisms and selectively block key molecules to provide certain guidelines for clinical targeted treatment of RB patients.

## Data Availability

All data generated or analyzed during this study are included in this published article.
